# The relative importance of competition and predation in environment characterized by resource pulses – an experimental test with a microbial community

**DOI:** 10.1186/1472-6785-13-29

**Published:** 2013-09-03

**Authors:** Teppo Hiltunen, Jouni Laakso

**Affiliations:** 1 Department of Food and Environmental Sciences / Microbiology and Biotechnology, University of Helsinki, P.O. Box 65, 00014 Helsinki, Finland; 2 Department of Biosciences / Integrative Ecology Unit, University of Helsinki, P.O. Box 65, 00014 Helsinki, Finland

**Keywords:** Community dynamics, Diversity, Predator–prey interaction, Resource competition, Resource pulse, *Tetrahymena thermophila*, Trophic dynamics

## Abstract

**Background:**

Resource availability and predation are believed to affect community dynamics and composition. Although the effects of resource availability and predation on prey communities are usually studied in isolation, these factors can also have interactive effects, especially since the outcome of competition under shared predation is expected to depend on resource availability. However, there are few experimental studies that test the interactive roles of resources and predation on dynamics of more complex multispecies communities. Here, we examine the importance of competition and predation on microbial community dynamics in a resource pulse environment.

**Results:**

We manipulated resource availability and predation simultaneously in a microbial microcosm experiment, where a bacterial community was exposed to the protozoan predator *Tetrahymena thermophila* in three different resource concentrations (low, intermediate and high). The prey community consisted of three heterotrophic bacterial species: *Bacillus cereus*, *Serratia marcescens* and *Novosphingobium capsulatum*, all feeding on a shared plant detritus medium. In fresh culture media, all species grew in all resource concentrations used. However, during experiments without any addition of extra resources, the existing resources were soon depleted to very low levels, slowing growth of the three bacterial species. Prior to the microcosm experiment, we measured the competitive ability and grazing resistance, *i.e.* reduced vulnerability to predation, of each prey species. The three species differed in allocation patterns: in general, *N. capsulatum* had the best competitive abilities and *B. cereus* had good grazing resistance abilities. In the long-term microcosm experiment, *N. capsulatum* dominated the community without predation and, with predation, *B. cereus* was the dominant species in the intermediate and high resource environments.

**Conclusions:**

Short-term, single-species assays revealed significant differences in the allocation of competitive and defensive traits among the prey species. Based on these differences, we were, to some extent, able to predict how the long-term community structure, e.g. species dominance, is modified by the resource availability and predation interaction in pulsed resource environments. Our results are consistent with theoretical predictions and also highlight the importance of interactive effects of resource competition and predation, suggesting that these factors should not be studied in isolation.

## Background

The resource competition theory predicts that competition will reduce species abundance and ultimately lead to dominance by a single species in cases where there is a constant supply of a single limiting resource [1]. However, resource availability is seldom constant in natural environments; instead, the frequency and magnitude of resource supply often varies resulting in the alternation of low and high resource availability [2,3]. Perhaps the most drastic changes in resource availability are caused by resource pulses. Resource pulses are defined as low frequency temporal events leading to increased resource availability with a short duration and a large magnitude [4]. The temporal variability in resource supply during resource pulse events makes predicting the outcome of resource competition and community dynamics more challenging. One way to approach this problem is to investigate the characteristics related to resource use among the individual species in the community. In general, a species’ ability to grow can differ between low and high resource environments so that the competitive dominance between species changes as a function of the resource concentration. In environments where resource pulses occur, rapid increases in resource concentrations can favor high resource specialists since their densities can respond rapidly to increasing resources. In contrast, long periods of low resource concentration can favor species that are able to maintain positive growth rates with very limited resources. Therefore, temporal changes in resource availability may allow different species to co-exist in the communities [1,5].

Predation, in addition to resource availability, is a major force shaping community structure. Predation often greatly reduces prey population density and alters community composition and species diversity [6,7]. Predation can have a positive effect on prey community diversity when predators feed more on superior competitors, which would, without predators, dominate the community [8-10]. Furthermore, many studies have shown that grazing resistance is often costly and the cost is seen as reduced competitive ability [11,12]. The effect of resources and predation on prey community is often linked. The effect of predation on prey community composition depends on resource availability [13-16]. Using a theoretical approach, Holt *et al.* (1994) found that traits related to resource competition are more important for prey fitness when resources are scarce. However, when resources are more abundant, traits related to grazing resistance can play a more significant role in prey fitness [16].

The aim of this study was to investigate the relative roles of resource competition and predation on prey community dynamics after a resource pulse. In an earlier study [17] with the same system, fluctuating resource supply and predation allowed different prey species to prevail under different fluctuation regimes through trade-offs between competitive ability and grazing resistance. The present study continued this work by investigating the community dynamics in an environment where resources do not fluctuate but are provided as a single large-scale pulse and are thereafter depleted by the bacterial community. We tested how the amount of resources (intensity of the resource competition) and predation by the protozoan predator *T. thermophila* affect the population dynamics and composition of an aquatic bacterial prey community. To answer these questions, we first conducted a short-term growth and feeding experiment to estimate the competitive ability and grazing resistance of each prey species. All measurements were conducted in three resource concentrations, low, intermediate and high. We used short-term population growth rate as an estimate of competitive ability and grazing resistance as an estimate of biomass reduction by predators. To investigate long-term community dynamics, we then conducted a 21-day long microcosm experiment where a three-species prey community was cultured in three resource concentrations with and without predators. During the experiment, we monitored population densities and prey community composition with direct microscope counts and a dilution plating method. The experimental set up simulated a resource pulse and we monitored the resultant community dynamics. We predicted that resources and predation should have a substantial effect on the prey community in the microcosm experiment, based on theory [6,7] and previous studies of similar microbial systems [9,10,17-19]. We also predicted that traits of individual species should explain community dynamics with traits related to competition being more important in the absence of predators and traits related to grazing resistance being more important when predators are present. In our case, we concluded, based on competitive ability and grazing resistance measurements, that *N. capsulatum* was the best resource competitor in low and intermediate resource concentrations and *B. cereus* had overall good grazing resistance abilities. The role of predation is considered to be less important in low resource environments where resource competition is more important [16]. One reason behind this prediction is that in less productive environments predator population sizes are also smaller and thus predation pressure is lower. The microcosm community experiment confirmed our predictions and the prey species that was a better resource competitor was dominant in the absence of the predator and the more grazing resistant species dominated in the presence of predation. Furthermore, the role of predation was less significant in the low resource environment where traits related to competitive ability seem to be more important in determining the identity of the dominant species.

## Results

For the simplified results summary see Table 1.

**Table 1:** Summary of the results from the trait and community experiments

	**Predator absent**		**Predator present**	
**Resource level**	**Best competitive ability**	**Dominant species in the community experiment**	**Best grazing resistance**	**Dominant species in the community experiment**
**Low**	*N. c*	*N. c*	*B. c / S. m*	*N. c*
**Intermediate**	*N. c*	*N. c*	*B. c / N. c*	*B. c*
**High**	*S. m*	*N. c*	*B. c / N. c*	*B. c*

Species codes: *B. cereus* (*B. c*), *S. marcescens* (*S. m*) and *N. capsulatum* (*N. c*).

### Trait measurements

#### Competitive ability

**Figure 1:**
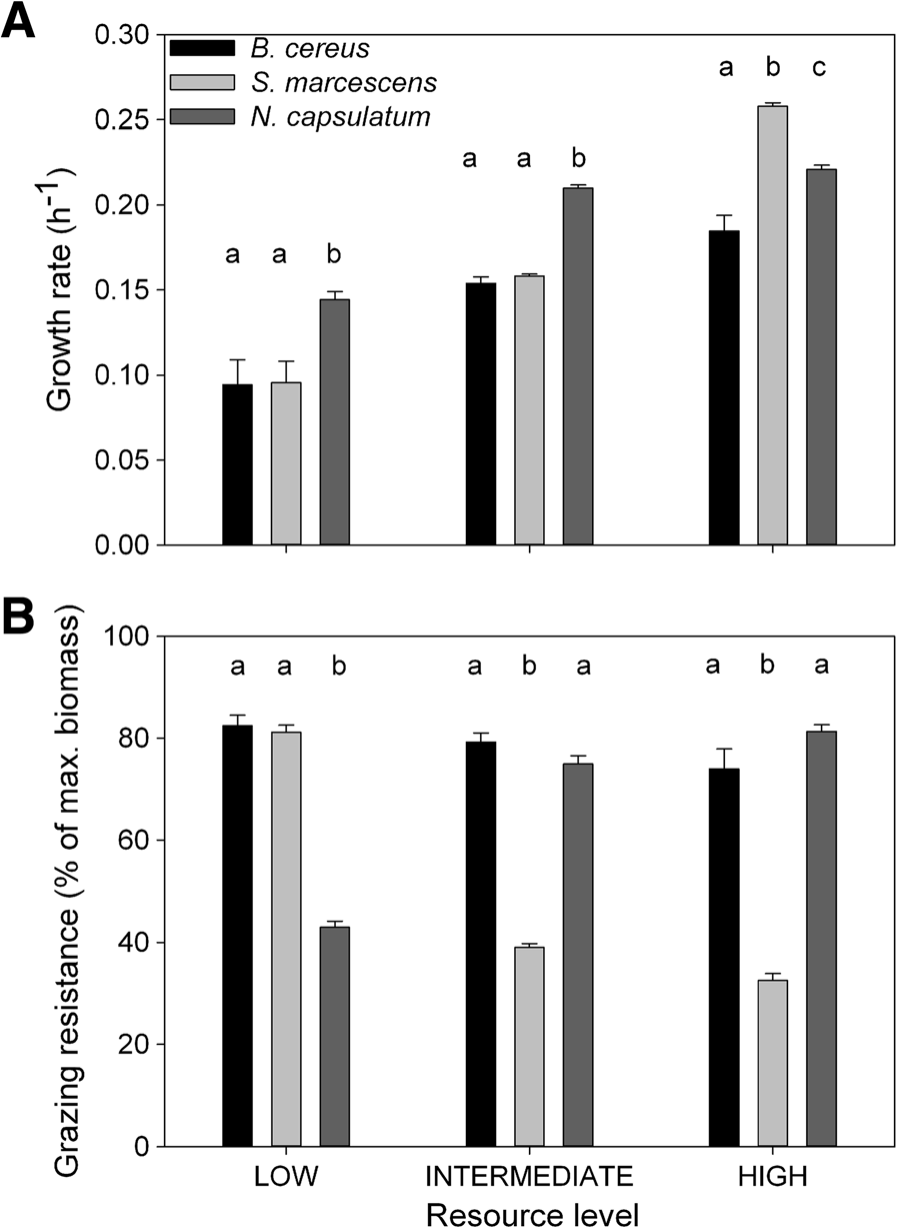
**Performance of the prey species in resource competition and predation environments.** Fitness measured as population growth rate **(A)** and grazing resistance **(B)** (mean ± S.E.). In both panels letters **(a**, **b** or **c)** indicate subsets that are statistically not different from each other.

Species growth rates increased with increasing resource concentration, but the competitive rank between species varied among resource concentrations (Figure 1A; resource treatment: F_2.98_ = 85.7; p < 0.0001, species identity × resource treatment: F_6.98_ = 36.7; p < 0.0001). We found that *N. capsulatum* had a higher growth rate than the two other species in low and intermediate resource concentrations when comparing species growth rates in different resource concentrations (Figure 1A; homogenous subsets; low resource concentration: a: p = 0.10; b: p = 1.00; intermediate resource concentration: a: p = 0.43; b: p = 1.00). *S. marcescens* had the highest growth rate, *N. capsulatum* had the second highest and *B. cereus* had the lowest, in high resource concentrations (Figure 1A; all species in different homogenous subsets).

#### Grazing resistance

Grazing resistance, as with population growth rates, was different among species and rank among species depended on the resource concentration (Figure 1B; species identity: F_3.98_ = 79.4; p < 0.0001, resource treatment: F_2.98_ = 19.5; p < 0.0001). The grazing resistance of *S. marcescens* decreased with increasing resource concentration, the grazing resistance of *N. capsulatum* increased, and resource concentration had no effect on the grazing resistance of *B. cereus* (Figure 1B, species identity × resource treatment: F_6.98_ = 75.2; p < 0.0001). We found that *N. capsulatum* had a lower grazing resistance in low resource concentrations than the two other species (Figure 1B; homogenous subsets; a: p = 0.84; b: p = 1.00). In intermediate and high resource concentrations, *S. marcescens* had lower grazing resistance than the other species (Figure 1B, homogenous subsets; intermediate resource concentration; a: p = 0.13; b: p = 1.00; high resource concentration; a: p = 0.12; b: p = 1.00).

### Community experiment

#### Population densities

**Figure 2:**
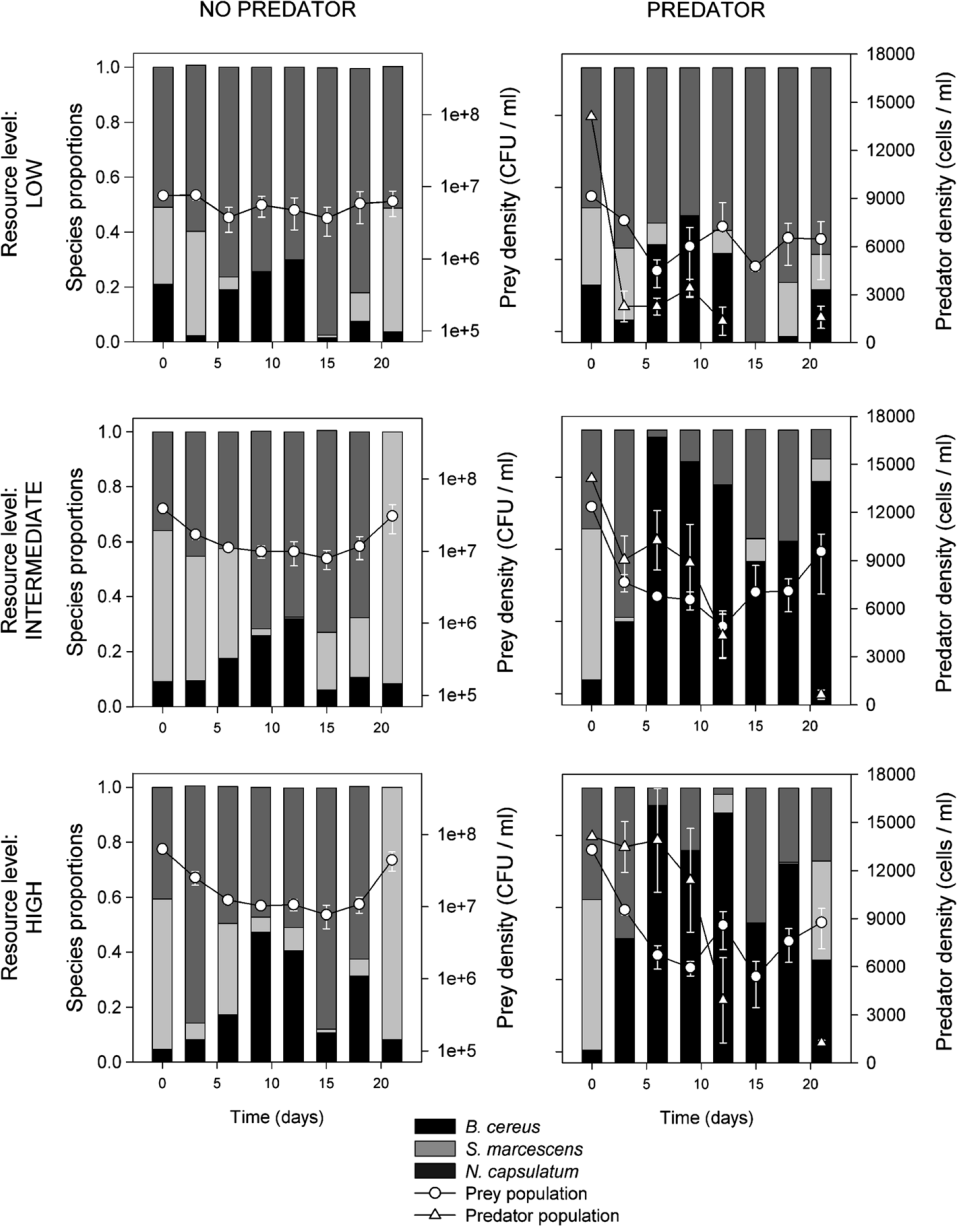
**The effects of experimental treatments (resource enrichment and predation) on community composition and population densities over time.** Vertical bars indicate species proportions in the prey community over time. Horizontal lines represent the total number of individuals in the prey community (circles, total number of prey individuals in the prey community) and *T. thermophila* population density (triangles) (mean ± S.E.).

The total number of prey individuals was lower when predators were present in all resource concentrations and each concentration produced low, intermediate and high population densities (Figure 2; predator treatment: F_1.18_ = 413; p < 0.0001, resource treatment: F_2.18_ = 440; p < 0.0001, all in different homogenous subsets). Predator population density was lower in low resource concentrations compared to intermediate and high resource concentrations (Figure 2; resource treatment: F_2.9_ = 30.1; p < 0.0001, intermediate and high resource concentrations in the same homogenous subset, p = 0.11). Furthermore, predator population density declined over time in all resource concentrations (time: F_1.9_ = 256; p < 0.0001).

#### Species proportions in the prey community

The effects of predation on prey community composition differed along the resource concentration axis. The proportion of *N. capsulatum* was higher than the two other species in the low resource concentrations, both with and without predators (Figure 2; predators absent: F_2.9_ = 58.8; p < 0.0001, *S.m* and *B.c* in the same homogenous subset, p = 0.93; predators present: F_2.9_ = 35.5; p < 0.0001, *S.m* and *B.c* in the same homogenous subset, p = 0.55). In intermediate resource concentrations, the proportions of all species were different from each other; the proportion of *N. capsulatum* was the highest in treatments without predators, *B. cereus* was the lowest, and the proportion of *B. cereus* in treatments with predators was the highest and *S. marcescens* the lowest (Figure 2; predators absent: F_2.9_ = 25.0; p < 0.0001, predators present: F_2.9_ = 63.8; p < 0.0001, in both, all species in different homogenous subsets). The proportion of *N. capsulatum* was higher than the other two species in high resource concentrations without predators (Figure 2; F_2.9_ = 15.2; p = 0.001, *S.m* and *B.c* in the same homogenous subset, p = 0.65). The proportion of *B. cereus* was the highest and *S. marcescens* was the lowest in high resource concentrations with predators (Figure 2; F_2.9_ = 116; p < 0.0001, all species in different homogenous subsets).

## Discussion

Competition for shared resources and predation are usually among the most important factors driving prey community dynamics [20]. To investigate the effects of these two factors on community dynamics after a resource pulse, we conducted a microbial microcosm experiment where we manipulated the intensity of resource competition with resource pulse concentration as well as the presence of predation. In order to predict and understand the outcome of community dynamics, we measured growth and defense related traits of each prey species prior to the long-term experiment. Based on these measurements, we were able to categorize *N. capsulatum* as a good resource competitor, *B. cereus* being more resistant against grazing and *S. marcescens* as an opportunist specialized in high resource environments (Figure 1). Results from the community experiment support this view: without predators in all resource concentrations and also with predators in low resource concentration, *N. capsulatum* was the dominant species (Figure 2). However, predation hindered the dominance of *N. capsulatum*; in intermediate and high resource concentrations with predation, the inferior competitor but more grazing resistant *B. cereus* was the dominant species (Figure 2). See Table 1 for a summary of the results.

Temporal changes in resource availability, such as resource pulses, are known to promote species coexistence [4]. This enhanced diversity is often explained by interspecific differences in the ability to maintain positive growth under low resource conditions and rapid growth when resources are abundant [21-24]. The role of fluctuating resources on species co-existence and community dynamics was tested in a previous study [17] using the same microbial community as here. The main finding in that study was that *S. marcescens* was the dominant prey species across all treatments [17], a qualitatively different result compared to the present study. However, in the Hiltunen *et al.* 2012 study, the inflow of fresh media, having the same concentration as “high” in the current study, was a frequent event (on average ~80% of the media was replaced daily), enabling *S. marcescens* to dominant the community*.* A likely reason why *S. marcescens* was able to dominate in the previous study was its high growth rate in the high resource concentration of the fresh culture media (Figure 1A: high resource concentration). In the current study, *S. marcescens* was not the dominant species in any of the treatments. *S. marcescens* seem to be a high resource specialist and in our current experiment, even in the initially high resource concentration, the availability of resources declined rapidly since there was no further input of new resources and the consumers depleted the existing resources. Thus, high resource specialists will experience a fitness benefit only for a short period of time and after a while species specialized in the use of low resource concentrations, such as *N. capsulatum* in our experiment, will dominate the communities. However, we found that in the intermediate and high resource concentrations without predators *S. marcescens* became the dominant species by the last time point and also increased its proportion substantially in the low resource environment during the last three time points. This is a result that cannot be explained with the competitive ability measurements. One explanation for this change in dominance after 21 days is the change in the resource quality. Pekkonen *et al.* (2012) studied resource mediated indirect facilitative and inhibitory interactions between *S. marcescens* and *N. capsulatum*, competing for the same plant detritus resource as used in our experiment. They found, among other things, that *S. marcescens* benefited from the presence of consumed growth media in contrast to *N. capsulatum*. To investigate similar questions, Lawrence *et al*. (2012) [25] used a nuclear magnetic resonance (NMR) spectroscopy to study the qualitative changes in a complex, organic plant based culture media. This approach could also have been useful in our study for investigating qualitative changes in our culture media and explain why *S. marcescens* was able to increase its proportion at the end of the experiment. However, based on data that we have now, we can conclude that in our resource pulse environment, temporal changes in the resource availability might not only have been quantitative but also qualitative. Even though we do not have direct evidence, this type of complex facilitative interaction could explain why the inferior competitor in low concentrations of fresh growth media could dominate at the end of the experiment when concentrations of the original resources were bound to be extremely low. However, this also makes predicting the competitive outcome based solely on growth rate measurements more challenging.

Predation is generally predicted to have a positive effect on coexistence among prey species when predators prevent the exclusion of more resistant but less competitive prey types [6,7,16,26]. The community data presented here is a good example of this scenario. Without predators, *N. capsulatum* was the dominant species; however, with predators in intermediate and high resource concentrations, the good resource competitor *N. capsulatum* was no longer able to dominate the prey community. *B. cereus*, an inferior resource competitor but an inherently more grazing resistant species, dominated the prey community in these conditions.

Interestingly, we found an interactive effect between resource concentration and predation treatments so that in low resource environments *N. capsulatum* was the dominant prey species instead of the more grazing resistant *B. cereus*. Holt *et al.* (1994) were the first to use a theoretical approach to investigate the interplay of exploitative and apparent competition and described the shift from dominance of exploitative to apparent competition along the enrichment gradient. Our experimental findings described above are consistent with the predictions by Holt *et al.* We observed, as the theory predicts, a shift in dominance from the superior resource competitor (*N. capsulatum*) to the grazing-resistant prey (*B. cereus*) along an enrichment gradient when the prey community was exposed to predation. Changes in the relative importance of competition and predation related traits along an enrichment axis might explain this finding in our experiments. When resources are extremely limited, traits related to competitive ability are disproportionally important and the importance of these traits may override any benefits that the higher grazing resistance can provide. Also, the fact that predator population density and, therefore, predation pressure was lower in the low resource environment could have contributed to making competitive ability a more important factor in determining the dominant prey species.

## Conclusions

We found that in our experimental bacterial communities, either resource availability or ciliate predation determined the dominant species. This is in line with theoretical predictions [1,2,6,7,21,23,26,27]. In most cases, we were able to predict the identity of the dominant species based on trait characteristics measured prior to the community experiment in single species short-term assays. We also found that resource concentration and predation treatments strongly interacted so that in low resource environments competitive ability was the main factor determining the community composition.

## Methods

### Study species and culture media

Our experimental prey community consisted of three heterotrophic bacterial species: *Bacillus cereus* (ATCC 14579), *Serratia marcescens* (ATCC 13880) and *Novosphingobium capsulatum* (ATCC 14666), all feeding on shared plant detritus medium. All strains were obtained from the American Type Culture Collection [28]. The criteria for selecting these three bacterial species included positive growth on both the nutrient broth agar and liquid prey culture medium and that the species were distinguishable based on their colony color and morphology. The source of organic carbon in our liquid prey culture medium was filtered plant detritus (hay extract, Ward’s natural science, Rochester, NY), for a detailed recipe and methods for culture media preparation see [17,18]. Three final concentrations of plant detritus used throughout experiment were 0.215, 1.075 and 2.15 mg/l and they are referred hereafter as low, intermediate and high resource concentrations. When the experiments were initiated, the bacterial strains (stored in 50% glycerol at −70°C) were first thawed and then cultivated on nutrient broth agar (10 g nutrient broth, 2.5 g yeast extract and 15 g agar (all Sigma-Aldrich) in 1 liter of dH_2_0.

As a predator, we used a ciliated protozoa, *Tetrahymena thermophila* (ATCC 30008), which is an asexual strain consisting of only a single mating type and has been widely used in experimental microbial ecology [8,9,17,29-31]*T. thermophila* was cultured in a proteose peptone-yeast medium (10 g nutrient broth, 2.5 g yeast extract (Sigma-Aldrich) in 1 liter of dH_2_0) in controlled laboratory conditions prior to the experiment. All experiments were carried out at 25 ± 1°C.

### Estimating competitive ability and grazing resistance

We conducted a short-term growth and feeding experiment to estimate the competitive ability and grazing resistance of each prey species. As an estimate of competitive ability, we used population growth rate and grazing resistance estimated as biomass reduction by predators. Measurements were conducted in each of the three resource concentrations (low, intermediate and high).

When measuring the population growth rates, 350 μl of fresh culture medium was inoculated with a small amount of bacteria (10 μl containing ~10^6 individuals). Then, the population was allowed to grow for 96 hours and the initial period when resources were not limiting was used to estimate the maximum growth rate (see below). When measuring grazing resistance, we used the same bacterial cultures that were allowed to reach carrying capacity (96 h) before adding 10 μl of centrifuged and washed predator stock in pH 7.5 phosphate buffer containing ~100 predator individuals. We monitored bacterial biomass (optical density) for another 96 hours which allowed predators to reach a high density and prey population was simultaneously grazed down. This reduction of prey biomass (percent grazed biomass from initial biomass prior to the addition of the predators) was then used as an estimate of the grazing resistance. All treatments were replicated nine times.

Population growth and predator-induced decline was measured with Bioscreen C spectrophotometer (Growth Curves AB Ltd, Finland) where optical density of each well was measured at 480–580 nm wavelengths at five minute intervals. Population growth rate was calculated as the slope of the linear regression of natural logarithms of population biomass versus time when the population grew at its maximal rate. This methodology in bacterial trait measurements has been used successfully in previous studies with similar systems (see e.g. [9,17,18]).

### Microcosm community experiment

To study community dynamics after a resource pulse, we conducted a factorial microcosm experiment in batch cultures where bacterial communities were cultured in three resource levels (low, intermediate and high), with and without predators. Each treatment was replicated four times.

The microcosms were contained in 250 ml polycarbonate Erlenmeyer flasks (Corning) and experimental communities were assembled as follows: each bacterial species was grown separately for 96 hours in liquid medium and then mixed in even proportions 10 ml each and 750 μl of centrifuged predator containing ~ 7.5 × 10^4 individuals was added to the predation treatments. At the beginning of the experiment, the total volume of liquid in each microcosm was 30.75 ml. The experiment was carried out for 21 days and sampling was conducted every three days by removing a 1 ml sample from each microcosm under sterile conditions.

A Lugol’s solution was used to fix 200 μl of the 1 ml sample and the predator population densities were determined automatically from the digitized images using an image recognition script (for detailed methods see [29]). Two predator samples, days 15 and 18, were misplaced and treated as missing data. The prey species population sizes were determined with serial dilution plating and by counting the colony forming units (CFU’s) cultured on petri dishes containing nutrient broth agar.

### Statistical analysis

The effect of resource concentration and predation on population growth rates and grazing resistance were analyzed with a two-way ANOVA. Post–hoc Tukey comparisons were performed to test pairwise interactions. In the community experiment, the effect of treatments on population densities and species proportions were analyzed with repeated measurements ANOVA (RMANOVA). The requirements of the RMANOVA only allowed the analysis of the first five samples of the predator population data due to the loss of samples from days 15 and 18. Population growth rates (h^-1^) were calculated as r = ln(N_t+1_/N_t_)/t where N_t_ is population size at time, t. All analyses were performed with PASW statistics (SPSS Inc. Chicago IL, v. 20.0) software.

## Abbreviations

CFU: Colony forming unit; ANOVA: Analysis of variance; RMANOVA: Repeated measures analysis of variance; B. c.: *Bacillus cereus*; S.m.: *Serratia marcescens*; N. c.: *Novosphingobium capsulatum.*

## Authors’ contributions

TH and JL designed the study. TH conducted the experiments, designed and performed the statistical analyses and drafted the manuscript. JL supervised the research and revised the manuscript. Both authors read and approved the final version of the manuscript.
